# Chemopreventive effects of a low-side-effect antibiotic drug, erythromycin, on mouse intestinal tumors

**DOI:** 10.3164/jcbn.16-107

**Published:** 2017-04-14

**Authors:** Takahiro Hamoya, Shingo Miyamoto, Susumu Tomono, Gen Fujii, Ruri Nakanishi, Masami Komiya, Shuya Tamura, Kyoko Fujimoto, Jiro Toshima, Keiji Wakabayashi, Michihiro Mutoh

**Affiliations:** 1 Epidemiology and Prevention Division, Research Center for Cancer Prevention and Screening, National Cancer Center, 5-1-1 Tsukiji, Chuo-ku, Tokyo 104-0045, Japan; 2 Department of Biological Science and Technology, Tokyo University of Science, 6-3-1 Niijuku, Katsushika-ku, Tokyo 125-8585, Japan; 3 Graduate Division of Nutritional and Environmental Sciences, University of Shizuoka, 52-1 Yada, Suruga-ku, Shizuoka 422-8526, Japan; 4 Division of Carcinogenesis and Cancer Prevention, National Cancer Center, 5-1-1 Tsukiji, Chuo-ku, Tokyo 104-0045, Japan; 5 Division of Molecular Biology, Nagasaki International University, 2825-7 Huis Ten Bosch, Sasebo, Nagasaki 859-3298, Japan

**Keywords:** erythromycin, Min mice, inflammation, oxidative stress, cancer chemoprevention

## Abstract

It is important to establish effective methods for preventing colorectal cancer because the number of colorectal cancer deaths is increasing. Erythromycin one of the macrolide antibiotics, has been shown to exert pleiotropic effects, such as anti-inflammatory and anti-oxidative effects, on mammalian cells. In the present study, we aimed to evaluate the preventive effects of erythromycin on intestinal carcinogenesis. We first confirmed that erythromycin suppresses the transcriptional activity of nuclear factor-κB and activator protein-1 and the expression of its downstream targets, interleukin-6 and cyclooxygenase-2 in human colon cancer cells. Next, we fed 5-week-old male *Apc* mutant Min mice with diets containing 500 ppm erythromycin for 15 weeks. Erythromycin treatment significantly reduced the number of proximal intestinal polyps to 70.9% of the untreated control value. Moreover, erythromycin reduced the levels of interleukin-6 and cyclooxygenase-2 mRNA expression in intestinal polyps. Although the levels of hepatic NADPH oxidase mRNA were decreased, erythromycin treatment did not affect the levels of oxidative stress markers, reactive carbonyl species, in the liver of Min mice. Our results suggest that erythromycin suppresses intestinal polyp development in Min mice, in part by attenuating local inflammation, and indicate that erythromycin is useful as a chemopreventive agent.

## Introduction

Colorectal cancer (CRC) is the most common cancer and the second-leading cause of cancer-related deaths in Japan. Thus, it is important to establish useful methods for preventing it. One method for preventing cancer entails using chemopreventive agents that meet the following criteria: i) they have convenient dosing schedules, ii) they are easily administered, iii) they are low cost, and, most importantly, iv) they have limited side effects.^(1)^

Macrolides are commonly used antibiotics that have good oral bioavailability and limited side effects, and exhibit prolonged tissue persistence. Macrolides bind to bacterial 50S ribosomes, not to eukaryotic cells, leading to the inhibition of transpeptidation or nascent peptide translocation, and thus cause a limited number of side effects. It has been reported that long-term low-dose use (400–600 mg/day for 6 months to 2 years) of erythromycin (EM), a macrolide antibiotic with a 14-membered ring, is effective against diffuse panbronchiolitis and does not cause severe side effects.^(2,3)^

Recently, EM has been reported to exert anti-inflammatory and anti-oxidative effects on mammalian cells *in vitro* and *in vivo*, in addition to exerting antimicrobial effects, by modulating the secretion of pro-inflammatory cytokines, such as interleukin (IL)-6, IL-1β and tumor necrosis factor (TNF)α,^(4)^ and inhibiting their oxidant burst by inhibiting neutrophil NADPH oxidase (NOX) activation.^(5)^ NOX is an enzyme that produces reactive oxides. EM has been reported to suppress mRNA expression and IL-8 release by inhibiting nuclear factor-κB (NF-κB) and activator protein-1 (AP-1) activity on bronchial epithelial cells.^(6)^ Of note, NF-κB and AP-1 dysregulation have been suggested to promote carcinogenesis.^(7–9)^ It has been established that both inflammation and oxidative stress status play pivotal roles in colorectal carcinogenesis. However, the preventive effects of erythromycin on intestinal carcinogenesis have not yet been elucidated.

In this study, we demonstrated that EM weakly but significantly suppressed NF-κB and AP-1 transcriptional activity and decreased intestinal tumorigenesis in Min mice, partly by modulating pro-inflammatory cytokine expression. In addition, EM administration reduced the mRNA expression levels of hepatic NADPH oxidase in Min mouse.

## Materials and Methods

### Chemicals

Erythromycin was purchased from Tokyo Chemical Industry (Tokyo, Japan). NF-κB inhibitor, 5HPP-33, was purchased from Merck KGaA (Darmstadt, Germany). Acrolein, crotonaldehyde, dansyl hydrazine (DH), glyoxal, 2,4-decadienal (DDE), heptadecanal, hexadecanal, 2,4-nonadienal (NDE), octadecanal, 2-octenal, pentadecanal, tetradecanal and 2-undecenal were also purchased from Tokyo Chemical Industry (Tokyo, Japan). Acetaldehyde, p-Toluenesulfonic acid (p-TsOH) and the reactive carbonyl species (RCs), including propanal, pentanal, butanal, 2-hexenal, hexanal, 2-heptenal, heptanal, octanal, 2-nonenal, nonanal, decanal, undecanal, dodecanal and tridecanal, were obtained from Sigma-Aldrich (St. Louis, MO). 4-Hydroxy-2-hexenal (HHE), 4-hydroxy-2-nonenal (HNE) and 4-oxo-2-nonenal (ONE) were purchased from Cayman Chemical Company (Ann Arbor, MI). p-Benzyloxybenzaldehyde (p-BOBA) was purchased from Wako Pure Chemical Industries (Osaka, Japan). 8-Heptadecenal (8-HpDE), 8,11-heptadecadienal (8,11-HpDDE) and 8,11,14-heptadecatrienal (8,11,14-HpDTE) were synthesized using a previously described method.^(10,11)^ Secosterol-A and B were synthesized according to a procedure reported by Wentworth *et al.*^(12)^ Stock solutions of the RCs and an internal standard (IS) (p-BOBA, 10 µM) were prepared separately in acetonitrile and stored at −20°C prior to use.

### Cell culture

HCT116 and SW48 cells, human colon adenocarcinoma cells, were purchased from the American Type Culture Collection (Manassas, VA). The HCT116 and the SW48 cells were maintained in DMEM supplemented with 10% heat-inactivated fetal bovine serum (FBS; Hyclone Laboratories Inc., Logan, UT) and antibiotics (100 µg/ml streptomycin and 100 U/ml penicillin) at 37°C with 5% CO_2_.

### Animals

Male C57BL/6-*Apc*^Min/^^+^ mice (Min mice) were purchased from Jackson Laboratory (Bar Harbor, ME). The mice (*n* = 3–4) were housed in plastic cages with sterilized softwood chips as bedding in a barrier-sustained animal room maintained at 24 ± 2°C and 55% humidity under a 12 h light/dark cycle. Erythromycin was mixed with an AIN-76A powdered basal diet (CLEA Japan, Inc., Tokyo, Japan) at concentrations of 0 and 500 ppm.

### Animal experiment protocols

Seven male Min mice aged 5 weeks were given 0 and 500 ppm erythromycin for 8 weeks. All animals housed in the same cage were included in the same treatment group. Food and water were available *ad libitum*. The animals were observed daily for clinical symptoms and mortality. Body weight and food consumption were measured weekly. At the sacrifice time point, the mice were anesthetized, and blood samples were collected from their abdominal veins. Their intestinal tracts were removed and separated into the small intestine, cecum and colon. The small intestine was divided into a proximal segment (4 cm in length) and the rest of the segment containing proximal (middle) and distal halves. The number of polyps in the proximal segments were counted and collected under a stereoscopic microscope.^(13,14)^ The remaining intestinal mucosa (non-polyp portion) was removed by scraping, and the specimens were stored at –80°C until quantitative real-time PCR analysis. The other regions were opened longitudinally and fixed flat between sheets of filter paper in 10% buffered formalin. Polyp numbers, size and intestinal distributions were assessed with a stereoscopic microscope. All experiments were performed according to the “Guidelines for Animal Experiments in the National Cancer Center” and were approved by the Institutional Ethics Review Committee for Animal Experimentation of the National Cancer Center. The animal protocol was designed to minimize pain or discomfort to the animals. The animals were acclimatized to laboratory conditions for more than two weeks prior to experimentation. All animals were euthanized by isoflurane overdose for tissue collection.

### Transient luciferase assays for AP-1 and NF-κB promoter transcriptional activity

To measure AP-1 and NF-κB promoter transcriptional activity, HCT116 and SW48 cells were seeded in 96-well plates (2 × 10^4^ cells/well). After 24-h incubation, the cells were transiently transfected with 200 ng/well pAP-1-Luc (Signosis Inc., Santa Clara, CA) or pGL4.32 [luc2P/NF-κB-RE/Hygro] (Promega, Madison, WI) reporter plasmid and 10 ng/well pGL4.73 [hRluc/SV40] control plasmid (Promega) using Polyethylenimine MAX MW 40,000 (PolyScience, Warrington, PA). The transfected cells were cultured for an additional 24 h and then treated with EM for 24 h. Firefly and Renilla luciferase activity levels were determined using the Bright GLO and Renilla GLO Luciferase Assay Systems (Promega), respectively. For the TNFα and IL-1β experiment, TNFα and IL-1β were administered at the same time as EM and NF-κB inhibitor of 5HPP-33. The luciferase activity percentages for each treatment were calculated from triplicate well data, and the values were normalized to those of Renilla luciferase activity. Data are expressed as the mean ± SD (*n* = 3).

### Luciferase assays for NF-κB promoter transcriptional activity in stable transfectants

To measure NF-κB transcriptional activity, HCT116 colon cancer cells were transfected with NF-κB-Luc (Promega) reporter plasmids using Polyethylenimine MAX MW 40,000 (PolyScience, Warrington, PA). The transfected cells were cultured for an additional 24 h. Cells stably expressing NF-κB-Luc were treated with hygromycin and cloned. These cells were referred to as HCT116-NF-κB-Luc cells. HCT116-NF-κB-Luc cells were seeded in 96-well plates (2 × 10^4^ cells/well). After a 24-h incubation, the cells were treated with EM and 10 ng/ml TNFα (Perotec, Somerset, NJ) for 24 h. Firefly luciferase activity levels were determined using the Bright GLO Luciferase Assay System (Promega). Basal NF-κB luciferase activity in the control was set as 1.0. Data are expressed as the mean ± SD (*n* = 4).

### Quantitative real-time polymerase chain reaction (PCR) analyses

Total RNA was isolated using RNAiso Plus (TaKaRa, Shiga, Japan) and 1 µg aliquots in a final volume of 20 µl were used for cDNA synthesis using a High Capacity cDNA Reverse Transcription Kit (Applied Biosystems, Foster City, CA). Real-time PCR was carried out using the CFX96/384 PCR Detection System (BIO RAD, Tokyo, Japan) and Fast Start Universal SYBR Green Mix (Roche Diagnostics, Mannheim, Germany), according to the manufacturers’ instructions. The primer sequences were as follows: IL-6 (5'-TGT TCT CTG GGA AAT CGT GGA and 5'-AAG TGC ATC ATC GTT GTT CAT ACA), COX2 (5'-GTG CCA ATT GCT GTA CAA GC and 5'-TAC AGC TCA GTT GAA CGC CT), NOX1 (5'-TCC CTT TGC TTC CTT CTT GA and 5'-CCA GCC AGT GAG GAA GAG TC), NOX2 (5'-GGG GTG TTG AAG GTC TCA AA and 5'-TGT TAC CAA CTG GGA CGA CA), p22^phox^ (5'-CGT GGC TAC TGC TGG ACG TT and 5'-TGG ACC CCT TTT TCC TCT TT), and glyceraldehyde-3-phosphate dehydrogenase (GAPDH) (5'-TTG TCT CCT GCG ACT TCA and 5'-CAC CAC CCT GTT GCT GTA). The following primers were used to evaluate human mRNA levels: human IL-6 (5'-CAC CCC TGA CCC AAC CAC AAA T and 5'-TCC TTA AAG CTG CGC AGA ATG AGA), human COX-2 (5'-GAT ACT CAG GCA GAG ATG ATC TAC CC and 5'-AGA CCA GGC ACC AGA CCA AAG A) and human GAPDH (5'-CCA CCC ATG GCA AAT TCC and 5'-TGG GAT TTC CAT TGA TGA CAA). To assess the specificity of each primer set, the melting curves of the amplicons generated by the PCR reactions were analyzed.

### Immunohistochemical staining

The middle segments of the small intestines were fixed, embedded, and sectioned as Swiss rolls for further immunohistochemical examination with the avidin–biotin complex immunoperoxidase technique. Polyclonal goat anti-COX-2 and anti-IL-6 antibody (Santa Cruz Biotechnology, Santa Cruz, CA) were used at 200× dilution. As the secondary antibody, biotinylated anti-goat IgG, absorbed with horse serum (Vector Laboratories, Burlingame, CA) were employed at 200× dilution. Staining was performed using avidin–biotin reagents (Vectastain ABC reagents; Vector Laboratories), 3,3'-diaminobenzidine and hydrogen peroxide, and the sections were counterstained with hematoxylin to facilitate orientation. As a negative control, consecutive sections were immunostained without exposure to the primary antibody.

### Extraction and analysis of reactive carbonyl species

Mouse liver (20 mg) samples were homogenized in 200 µl of sodium phosphate buffer (50 mM, pH 7.4) containing 0.5 mM EDTA and 20 µM BHT. The liver homogenates were mixed with an IS (p-BOBA) (20 pmol) and 400 µl of a chloroform/methanol (2:1, v/v) solution. The resulting mixture was vigorously agitated for 1 min and then centrifuged at 15,000 rpm for 10 min, and the organic phase was collected. The remaining precipitate and aqueous phases were then mixed with 400 µl of the chloroform/methanol solution (2:1, v/v), and the resulting mixture was centrifuged at 15,000 rpm for 10 min to obtain the organic phase. The combined organic phases were mixed with 100 µl of acetonitrile containing 50 µg of DH and 10 µg of p-toluenesulfonic acid and incubated for 4 h at ambient temperature in the absence of light. The mixtures were then evaporated to dryness *in vacuo* to yield the corresponding derivatized residues. These residues were dissolved in 200 µl of acetonitrile, and 5 µl of each sample was injected into the LC/MS system. The details regarding RCs analysis by LC/MS were described previously.^(15,16)^

### Statistical analyses

All results are expressed as the mean ± SD, and all statistical analyses were performed using Student’s *t* tests, with the exception of the analyses of AP-1 and NF-κB promoter transcriptional activity in HCT116 cells, which were performed using Dunnett’s test. Differences were considered statistically significant at *p<0.05, **p<0.01 and ***p<0.001.

## Results

### Suppression of AP-1 and NF-κB promoter transcriptional activity by erythromycin

**Fig. 1:**
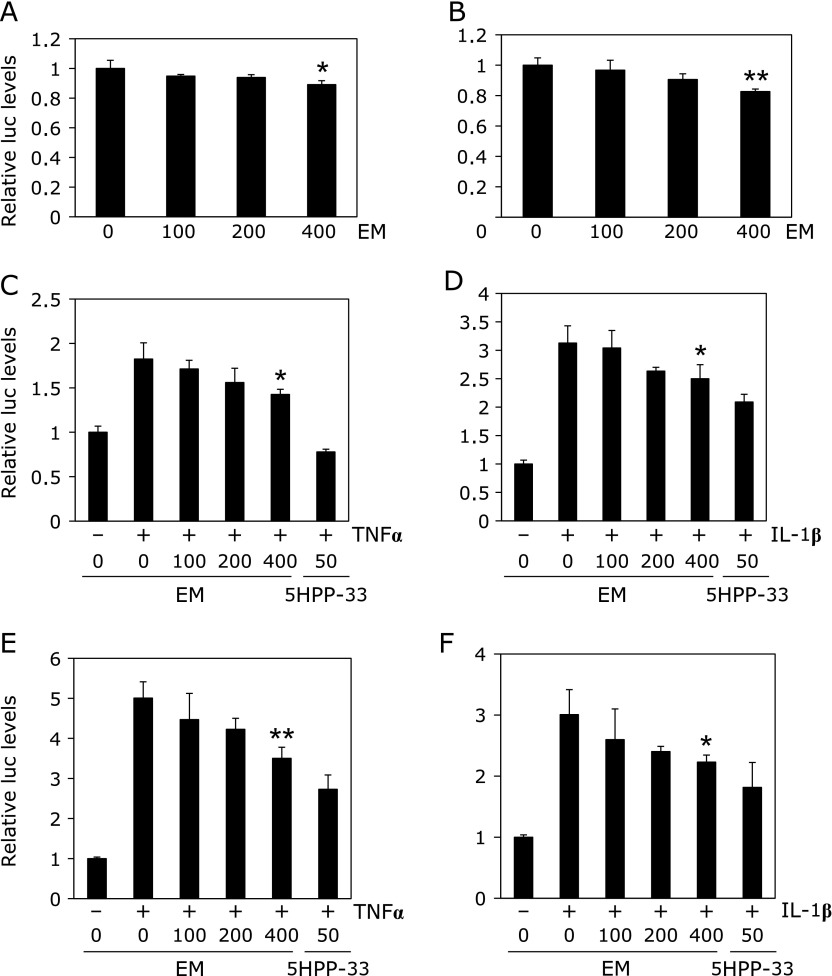
Effect of erythromycin on AP-1 and NF-κB promoter transcriptional activity in HCT116 and SW48 cells. HCT116-NF-κB-Luc cells were treated with erythromycin for 24 h (A). After transient AP-1 reporter plasmid transfection for 24 h, HCT116 cells were treated with EM for 24 h. AP-1 promoter transcriptional activity after 100, 200 and 400 µM EM treatment for 24 h (B). In HCT116 cells, TNFα-induced (C) and IL-1β-induced (D) NF-κB promoter transcriptional activity after 100, 200 and 400 µM EM treatment for 24 h. In SW48 cells, TNFα-induced (E) and IL-1β-induced (F) NF-κB promoter transcriptional activity after 100, 200 and 400 µM EM treatment for 24 h. The basal luciferase activity level of the control was set as 1.0. Data are the mean ± SD, *n* = 3. *p<0.05, **p<0.01 vs 0 control. TNFα: 10 ng/ml. IL-1β: 10 ng/ml. 5HPP-33: 50 µM used as a positive control.

AP-1 and NF-κB promoter transcriptional activities were examined following 24 h of EM treatment (100, 200 and 400 µM) in HCT116 cells and SW48 cells (Fig. 1). We confirmed the above findings regarding NF-κB promoter transcriptional activity using a stable transfectant, HCT116-NF-κB-Luc cells. Twenty-four hours of 400 µM EM treatment decreased NF-κB promoter transcriptional activity by 11% (*p*<0.05), compared with the untreated control value (Fig. 1A). Furthermore, twenty-four hours of 100, 200 and 400 µM erythromycin treatment decreased TNFα-stimulated NF-κB promoter transcriptional activity by 14% (*p*<0.05), 17% (*p*<0.05) and 21% (*p*<0.01), respectively, compared with the untreated control value (Supplemental Fig. 1A*).

In HCT116 cells, EM treatment slightly decreased AP-1 and NF-κB promoter transcriptional activities in a dose-dependent manner (Fig. 1B and Supplemental Fig. 1B). Twenty-four hours of 400 µM EM treatment decreased AP-1 and NF-κB promoter transcriptional activity by 18% (*p*<0.01) and 29% (*p*<0.01), respectively, compared with the untreated control value. Even in cells stimulated with 10 ng/ml TNFα and 10 ng/ml IL-1β, EM decreased NF-κB transcriptional activity in a dose-dependent manner (Fig. 1C and D). NF-κB inhibitor, 5HPP-33, was used as a positive control. Twenty-four hours of 400 µM EM treatment decreased TNFα-stimulated and IL-1β-stimulated NF-κB promoter transcriptional activity by 21% (*p*<0.05) and 22% (*p*<0.05) compared with the untreated control value, respectively.

In other colorectal cancer cells, SW48 cells, EM treatment slightly decreased TNFα-stimulated or IL-1β-stimulated NF-κB promoter transcriptional activities in a dose-dependent manner (Fig. 1E and F). Twenty-four hours of 400 µM EM treatment decreased TNFα-stimulated and IL-1β-stimulated NF-κB promoter transcriptional activity by 26% (*p*<0.05) and 31% (*p*<0.01) compared with the untreated control value, respectively.

### Suppression of inflammation-related factor mRNA expression in human colon cancer cells by erythromycin

**Fig. 2:**
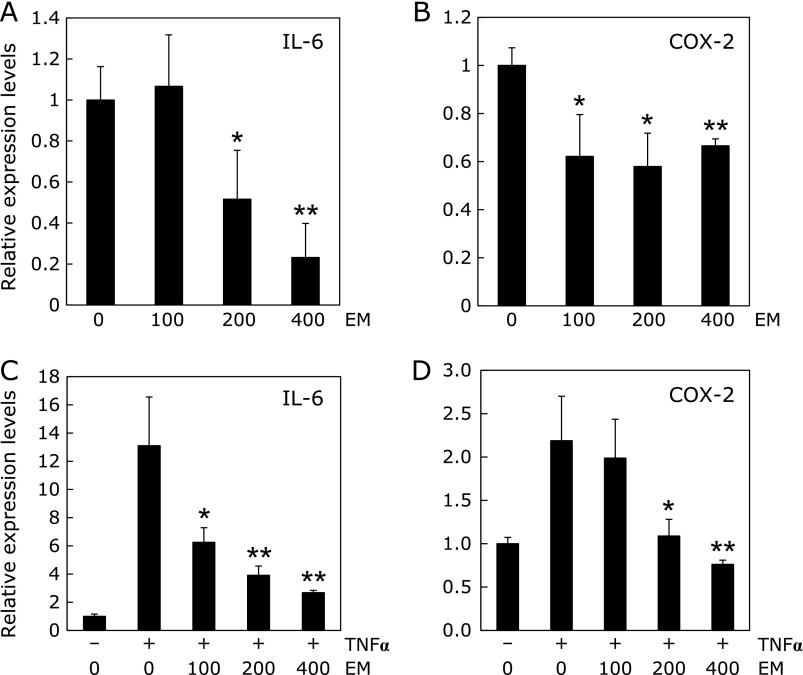
mRNA expression levels of inflammation-related factors in human colon cancer cells treated with or without erythromycin. HCT116 cells were seeded in 24-well plates at a density of 2 × 10^5^ cells well and cultured in medium containing 100, 200 and 400 µM EM for 24 h. After 24 h of treatment, quantitative real-time PCR analysis was performed to determine IL-6 (A) and COX-2 mRNA expression levels (B). Furthermore, HCT116 cells were treated with 10 ng/ml TNFα and EM for 24 h to determine the effects of these treatments on IL-6 (C) and COX-2 mRNA expression levels (D). The basal mRNA expression levels of the control were set as 1.0. Data were normalized with GAPDH. Data are the mean ± SD, *n* = 3. *p<0.05, **p<0.01, vs 0 ppm with 10 ng/ml TNFα.

To clarify the effects of EM on the downstream targets of AP-1 and NF-κB, inflammation-related factor mRNA expression was evaluated in HCT116 cells following 24 h of 100, 200 and 400 µM EM treatment with and without 10 ng/ml TNFα treatment. EM significantly suppressed IL-6 and COX2 mRNA expression with and without TNFα treatment (Fig. 2).

### Suppression of intestinal polyp formation in Min mice by erythromycin

Administration of 500 ppm EM to Min mice for 8 weeks did not affect body weight, food intake or clinical symptoms throughout the experimental period. There was no difference in average daily food intake between the 0 and 500 ppm groups of Min mice. In addition, no changes in organ weights that may have been attributable to toxicity were observed. Table 1 summarizes the data regarding the numbers and distributions of intestinal polyps in the basal diet control group and EM-treated group. The majority of polyps developed in the small intestine, while only a few developed in the colon. Treatment with 500 ppm EM decreased the total number of polyps to 72.6% of the untreated control value. A reduction in the number of polyps to 70.9% of the untreated control value was observed in the proximal segment of the small intestine (*p*<0.01 vs control group). No significant differences in the numbers of polyps were observed in the other segment of the small intestine or the colon following EM treatment. Supplemental Fig. 2* shows the size distributions of the intestinal polyps in the basal diet and EM-treated groups. The majority of polyps were approximately 3.0 mm in diameter. EM treatment significantly reduced the numbers of polyps <0.5 mm in diameter (*p*<0.05 vs control group).

### Suppression of inflammation-related gene expression in the intestinal polyps of Min mice by erythromycin

**Fig. 3:**
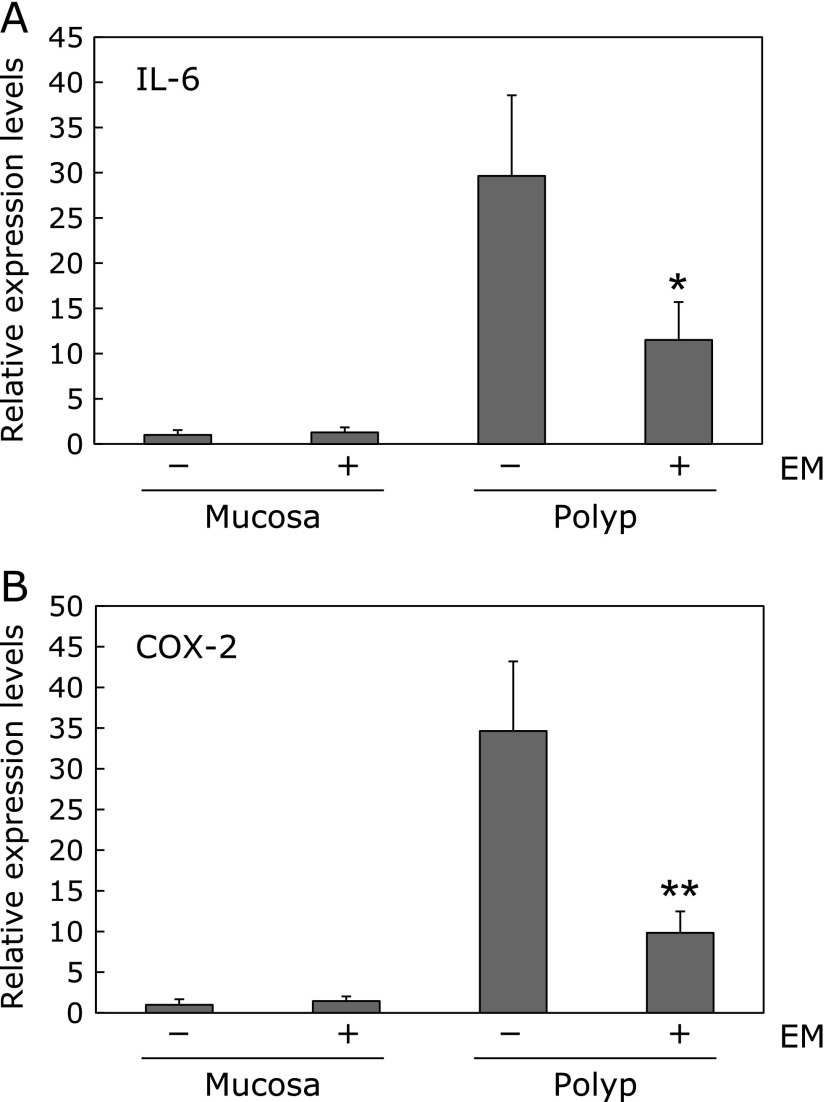
mRNA expression levels of inflammation-related factors in the intestines of Min mice. Quantitative real-time PCR analysis was performed to determine IL-6 mRNA (A) and COX-2 mRNA (B) expression levels in the non-polyp (mucosa) and polyp portions of the intestines of Min mice. Data were normalized with GAPDH. Each expression level in the non-polyp portions of the intestines in the control group (0 ppm) was set as 1. Data are the mean ± SD, *n* = 4. *p<0.05, **p<0.01, vs control group.

 To clarify the mechanisms underlying EM-mediated suppression of intestinal polyp formation, inflammation-related gene expression in the non-polyp (mucosa) and polyp portions of the intestine was investigated (Fig. 3). Real-time PCR revealed that treatment with 500 ppm EM for 8 weeks significantly suppressed IL-6 and COX-2 mRNA expression in the intestinal polyp segments by 38% (*p*<0.05) and 28% (*p*<0.01), respectively, compared with the untreated control values, respectively. In the non-polyp (mucosa) portion, no difference of IL-6 and COX-2 mRNA expression between the EM treatment and non-treatment group was observed.

### Localization of inflammation-related molecules expression in the intestinal polyps of Min mice

To confirm the localization of IL-6 and COX-2 in the polyp portions of the intestine, expression of IL-6 and COX-2 were examined by immunohistochemistry. IL-6 was observed mainly in the surface area of the cytoplasm of epithelial cells, and COX-2 was observed strongly in the stroma cells in the intestinal polyp of untreated Min mice. (Supplemental Fig. 3*).

### EM decreased NADPH oxidase mRNA expression in hepatic, but not in intestinal in Min mice

**Fig. 4:**
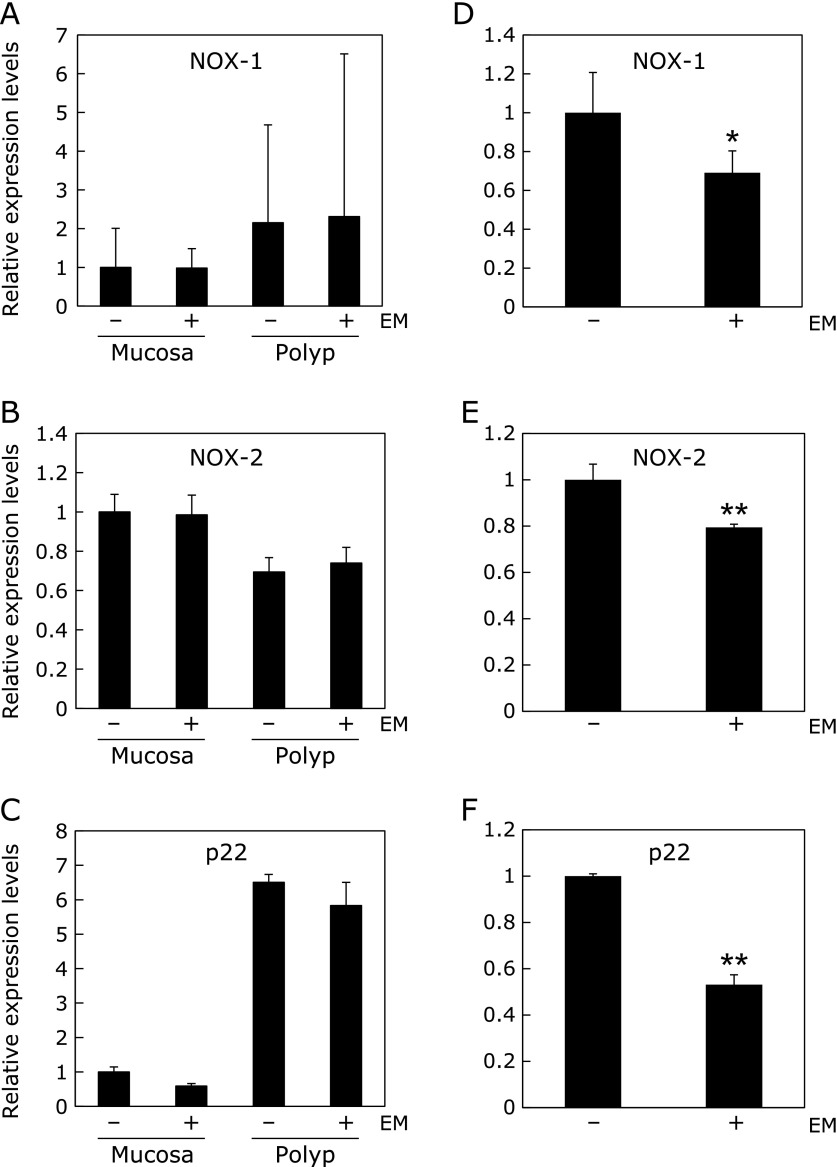
NADPH oxidase mRNA levels in the intestines and livers of Min mice. Quantitative real-time PCR analysis was performed to determine NOX-1 (A), NOX-2 (B) and p22^phox^ (C) mRNA expression levels in the intestines of Min mice. Moreover, quantitative real-time PCR analysis was performed to determine NOX-1 (D), NOX-2 (E) and p22^phox^ (F) mRNA expression levels in the livers of Min mice. Data were normalized with GAPDH. Each expression level in the livers of the untreated group was set as 1. Data are the mean ± SD, *n* = 4. *p<0.05, **p<0.001, vs 0 ppm.

We attempted to elucidate the oxidative stress-related mechanisms underlying the suppression of intestinal polyp formation. We first examined components of NADPH oxidase expression in the non-polyp (mucosa) and polyp portions of the intestine and found that NOX-1, NOX-2 and p22^phox^ mRNA expression was unchanged (Fig. 4A–C). We then examined the effects of EM on liver NADPH oxidase expression because we could not rule out the possibility that general oxidative stress may affect local intestinal polyp development. Real-time PCR revealed that 500 ppm EM treatment for 8 weeks significantly suppressed NOX-1, NOX-2 and p22^phox^ mRNA expression in the liver by 31% (*p*<0.05), 21% (*p*<0.001) and 47% (*p*<0.001), respectively, compared with untreated control values (Fig. 4D–F).

### Some decreases in the levels of oxidative stress-related markers in Min mice treated with erythromycin

**Fig. 5:**
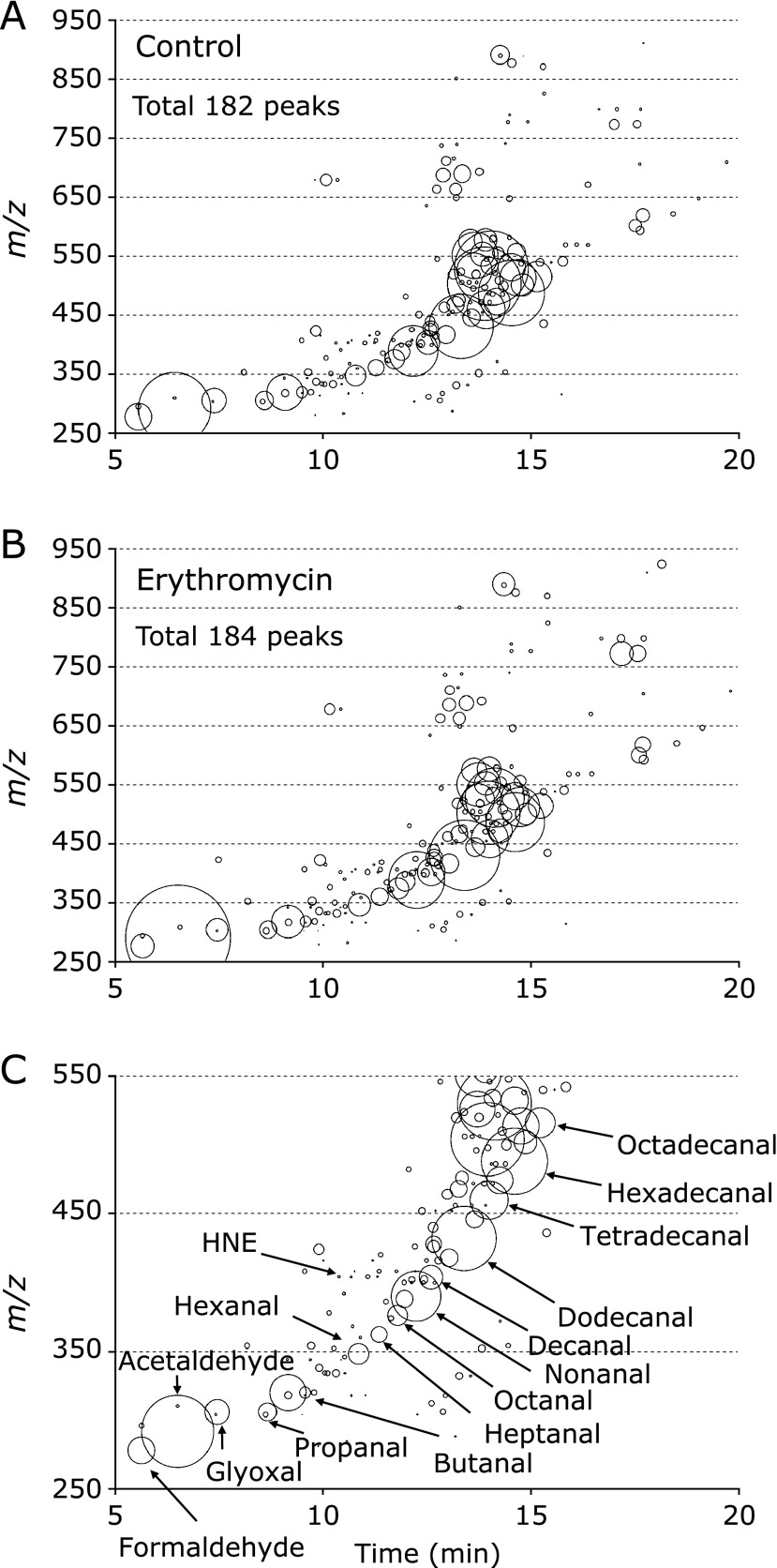
Corresponding RCs maps plotting free RCs detected in the liver samples of Min mice. All free RCs detected in the liver samples taken from 0 (A) or 500 (B) ppm EM-treated Min mice are shown. The RCs are plotted as circles as a function of their retention times (horizontal axis) and m/z values (vertical axis). The areas of the circles represent the intensities of the peaks of the RCs relative to that of the IS. Fig. 5C is an enlarged view of Fig. 5B showing the m/z values within the range of 250 to 550, as well as the names of specific RCs identified by the analyses. The RCs abbreviations are listed in Materials and Methods.

**Fig. 6:**
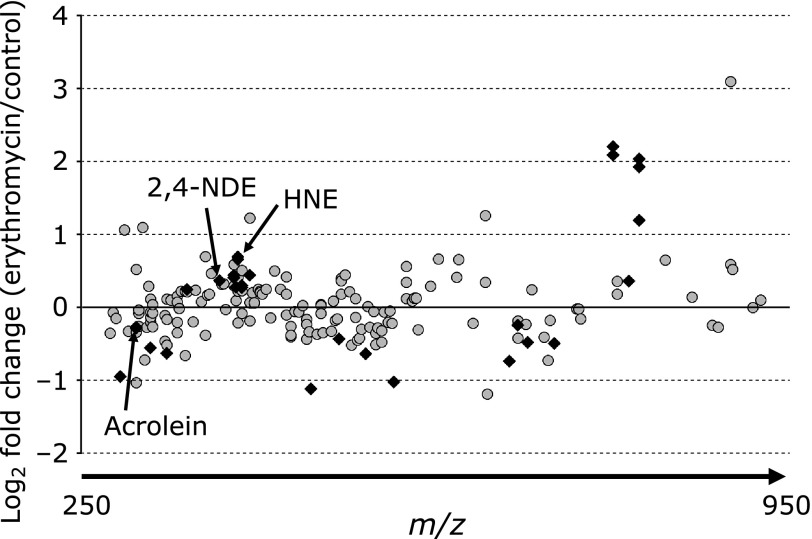
Relative RCs levels detected in the liver samples taken from erythromycin-treated Min mice compared with those detected in the liver samples taken from non-treated mice. The comparative RCs profiles of the liver samples from 500 ppm EM-treated Min mice. The closed diamonds indicate that the RCs levels were significantly different between non-treated and EM-treated mice (*p*<0.05).

To confirm the effects of EM on oxidative stress, we used liquid chromatography/mass spectrometry (LC/MS) and measured the levels of RCs in the livers of Min mice treated with or without EM. LC/MS detected 182 and 184 peaks in the samples taken from non-treated and 500 ppm EM-treated Min mice, respectively (Fig. 5). Of the 184 peaks detected in the samples taken from EM-treated Min mice, 90 tended to have lower levels and 74 tended to have higher levels, compared to the peaks detected in the samples taken from non-treated Min mice. Moreover, 13 were at significantly lower levels and 17 were at significantly higher levels, compared to the peaks detected in the samples taken from non-treated Min mice (Fig. 6, Table 2). Thus, EM administration did not affect the levels of RCs in the liver of Min mice.

## Discussion

In the present study, we assessed the effectiveness of EM as a colorectal cancer chemopreventive agent by evaluating its ability to suppress inflammation- and oxidative stress-related transcription factor activities. We then confirmed that EM weakly inhibited intestinal polyp development in Min mice. We also confirmed that the expression levels of the downstream targets of NF-κB and AP-1, such as IL-6 and COX-2, were decreased in the polyp portions of the intestine. However, EM administration also reduced the mRNA expression levels of hepatic NADPH oxidase.

We investigated the effects of EM on NF-κB and AP-1 transcriptional activity because both transcriptional factors play an important role in colorectal carcinogenesis. Moreover, activation of both NF-κB and AP-1 induce the expression of inflammatory cytokines, growth factors and inflammation-related enzymes, such as COX-2 and IL-6. Both COX-2 and IL-6 have been shown to play important roles in colorectal carcinogenesis.^(17,18)^ In our *in vitro* study using HCT116 cells, EM treatment clearly suppressed COX-2 and IL-6 mRNA expression in both the presence and the absence of TNFα stimulation. However, its weak inhibition of NF-κB and AP-1 transcriptional activity does not explain its suppression of COX-2 and IL-6 mRNA expression. We surmised that both the IL-6 and COX-2 genes possess a NF-κB and AP-1 responsible element^(19,20)^ in their promoter region, and additional activation of the responsible element could synergistically enhance resultant gene expression. For example, it has been reported that RR-ARFs inhibit both NF-κB and AP-1 transcriptional activity, and both inhibitions synergistically suppress COX-2 and IL-6 mRNA expression in RAW264.7 cells.^(21)^ Of course, we cannot exclude other possibilities regarding the independence of IL-6/ COX-2 and NF-κB/AP-1 transcriptional activity at this point.

Of note, it has been reported that EM (0.1–1,000 nM) suppressed IL-1β-stimulated synovial cells expressing COX-2 mRNA and protein via p38 MAPK phosphorylation, but NF-κB DNA binding activity was not observed at this dose.^(22)^ The effects of EM on NF-κB transcriptional activity should be confirmed using several cell lines in the future.

It has been reported that inflammatory factors such as COX-2 are up-regulated in stroma cells in intestinal polyps/adenomas of *Apc*-mutant mice. However, when they transform to adenocarcinomas, the expression of COX-2 is apparent in the epithelial cells of a tumor. As we showed in the immunohistochemical assay, the expression of COX-2 was apparent in the stroma cells in the intestinal polyps/adenomas of *Apc*-mutant mice (Supplemental Fig. 3*). IL-6 was observed mainly in the surface area of the cytoplasm of epithelial cells in intestinal polyps. Taking into consideration that inflammatory factors such as IL-6 or prostaglandine E_2_ (metabolites of COX-2) may act through paracrine communications, reduction of IL-6 or prostaglandine E_2_ production may play an important role in carcinogenesis, especially in the early stage of carcinogenesis.

COX-2 and IL-6 mRNA expression was clearly suppressed in the polyp portions of the intestines of Min mice, which may have affected local tumor growth and may explain the decreases in polyp numbers elicited by EM treatment.

To the best of our knowledge, this is the first report showing that EM can suppress intestinal polyp formation in mice. Some other macrolides are known to have anti-cancer effects and are part of a group of anti-cancer chemotherapy drugs classified as “antitumor antibiotics.” For instance, mitomycin-C is used in the treatment of many cancers, such as anal, bladder, breast, cervical, colorectal, head-and-neck, and non-small cell lung cancer. Rapamycin is used to treat renal cell cancer, neuroendocrine tumors and metastatic breast cancer by inhibiting mammalian target of rapamycin (mTOR) activation.^(23)^ Bryostatin and epothilone have also been used to treat cancer, and many other macrolides, such as exiguolide, are under investigation.^(24)^ Among macrolides, EM has the fewest side effects.

EM treatment tends to reduce polyp development in the colon and small intestine, but only significantly in the proximal portion of the small intestine. We were unable to determine in this study why this was so. Other agents as well, such as LPL inducers NO-1886 and PPAR ligands, have been shown to significantly reduce the number of intestinal polyps in the proximal part of the intestine.^(25,26)^ On the other hand, indomethacin, a COX inhibitor; nimesulide, a COX-2 selective inhibitor; sesamol, a COX-2 suppressor; and apocynin, an NADPH oxidase inhibitor, have been shown to mainly reduce the numbers of intestinal polyps in the middle to distal parts of the small intestine.^(27–30)^ We surmised that localized EM absorption or some unknown EM functions played a role in its effects on proximal intestinal polyp development.

We also analyzed the expression of NADPH oxidase (NOX) components, such as NOX1, NOX2 and p22^phox^, in the liver and intestine, as EM has been reported to inhibit neutrophil NOX activation, and the NADPH oxidase inhibitor apocynin has been reported to suppress intestinal polyp development in Min mice.^(30)^ Interestingly, the expression of hepatic NOX1, NOX2 or p22^phox^ but not intestinal NOX1, NOX2 or p22^phox^, was suppressed in the group treated with EM. Thus, we evaluated the levels of oxidative stress markers, RCs, in the liver of EM-treated Min mice. However, EM administration did not affect the levels of the liver RCs. Thus, it is not conclusive whether EM suppresses oxidative stress or not.

In conclusion, this study demonstrated that EM suppresses the development of intestinal polyps in Min mice, in part by attenuating local inflammation. Our findings suggest that EM is a useful chemopreventive agent in the aspect of drug repositioning.

## Figures and Tables

**Table 1:** The number of intestinal polyps/mouse in Min mice treated with or without erythromycin

Erythromycin (ppm)	No. of mice	No. of polyps/mouse				
		Small intestine			Colon	Total
		Proximal	Middle	Distal		
0	7	7.9 ± 4.3	20.3 ± 5.6	35.9 ± 16.1	0.7 ± 1.0	64.7 ± 21.3
500	7	2.3 ± 1.4*	17.7 ± 5.2	28.3 ± 11.2	0.1 ± 0.4	47.0 ± 13.5

Data are presented as the means ± SD. Significantly different from the untreated control group at *p<0.01.

**Table 2:** The levels of RCs detected in the liver (pmol/mg wet tissue)

Compounds	Control	Erythromycin
Acetaldehyde	36,657.2 ± 7,070.5	76,880.8 ± 82,777.6
Acrolein	1,165.5 ± 125.7	947.3 ± 116.7**
Glyoxal	12,360.8 ± 1,624.5	10,380.5 ± 2,136.9
Propanal	5,919.6 ± 488.3	5,606.8 ± 1,121.5
Crotonaldehyde	1,491.8 ± 223.7	1,301.3 ± 236.5
Butanal	3,157.6 ± 385.8	3,005.2 ± 426.6
Pentanal	671.1 ± 81.8	728.6 ± 120.5
2-Hexenal	156.3 ± 26.5	155.2 ± 35.3
Hexanal	8,403.2 ± 1,651.6	9,859.4 ± 1,288.6
2-Heptenal	110.2 ± 27.2	97.8 ± 18.9
Heptanal	2,125.4 ± 730.4	2,508.1 ± 718.7
2-Octenal	81.2 ± 12.4	91.0 ± 12.7
Octanal	4,857.5 ± 1,324.8	5,532.1 ± 1,051.9
2,4-NDE	299.8 ± 37.8	385.9 ± 45.3*
2-Nonenal	1,043.5 ± 98.0	1,306.1 ± 270.5
Nonanal	13,480.2 ± 3,013.7	16,970.2 ± 2,868.9
2,4-DDE	239.7 ± 27.0	326.6 ± 80.9*
HNE	217.5 ± 47.1	341.5 ± 75.7**
Decanal	2,169.8 ± 674.4	2,897.8 ± 723.2
2-Undecenal	75.6 ± 10.3	104.0 ± 15.8***
Undecanal	1,254.8 ± 256.5	1,450.8 ± 319.4
Dodecanal	19,945.8 ± 5,636.9	23,770.4 ± 3,946.5
Tridecanal	2,362.8 ± 833.7	2,820.3 ± 857.4
Tetradecanal	1,319.5 ± 359.0	1,275.9 ± 386.1
Pentadecanal	5,275.3 ± 2,039.9	4,198.6 ± 2,320.0
Hexadecanal	9,318.7 ± 2,994.8	7,359.1 ± 2,736.5
8,11,14-HpDTE	261.6 ± 76.5	213.3 ± 81.1
8,11-HpDDE	349.3 ± 141.2	373.4 ± 88.9
8-HpDE	329.9 ± 34.3	291.3 ± 127.0
Heptadecanal	1,075.1 ± 401.5	969.1 ± 372.1
Octadecanal	2,176.8 ± 1,025.5	1,532.6 ± 773.6

Data are mean ± SD (*n* = 5). Significantly different from the control untreated group at *p<0.05, **p<0.01, ***p<0.001.

## Abbreviations

AP-1: activator protein-1
COX: cyclooxygenase
CRC: colorectal cancer
EM: erythromycin
FBS: fetal bovine serum
GAPDH: glyceraldehyde-3-phosphate dehydrogenase
IL: interleukin
IS: internal standard
LC/MS: liquid chromatography/mass spectrometry
NF-κB: nuclear factor-κB
NOX: NADPH oxidase
PCR: polymerase chain reaction
RCs: reactive carbonyl species
SRM: selected reaction monitoring
TNF: tumor necrosis factor
