# Supplementary material for: Chemopreventive effects of a low-side-effect antibiotic drug, erythromycin, on mouse intestinal tumors
Source: J Clin Biochem Nutr. 2017 Apr 14;60(3):199–207. doi: 10.3164/jcbn.16-107 (PMC5453017; doi:10.3164/jcbn.16-107)
Supplement: Supplemental Fig. 1 [file jcbn16-107sf01.pdf]

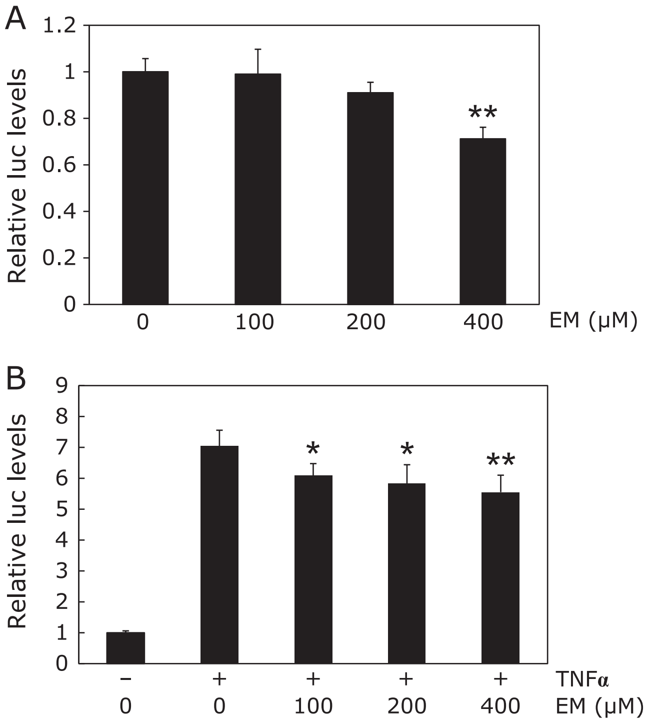

Supplemental Fig. 1. After transient NF-κB reporter plasmid transfection for 24 h, HCT116 cells were treated with EM for 24 h. NF-κB promoter transcriptional activity after 100, 200 and 400 μ pEM treatment for 24 h (A). HCT116-NF-κB-Luc cells were treated with erythromycin and 10 ng/ml TNFα for 24 h (B). The basal luciferase activity of the control was set as 1.0. Data are mean ± SD, *n* = 4. **p*<0.05, ***p*<0.01 vs 0 ppm with 10 ng/ml TNFα. EM: erythromycin.
