# Supplementary material for: Chemopreventive effects of a low-side-effect antibiotic drug, erythromycin, on mouse intestinal tumors
Source: J Clin Biochem Nutr. 2017 Apr 14;60(3):199–207. doi: 10.3164/jcbn.16-107 (PMC5453017; doi:10.3164/jcbn.16-107)
Supplement: Supplemental Fig. 2 [file jcbn16-107sf02.pdf]

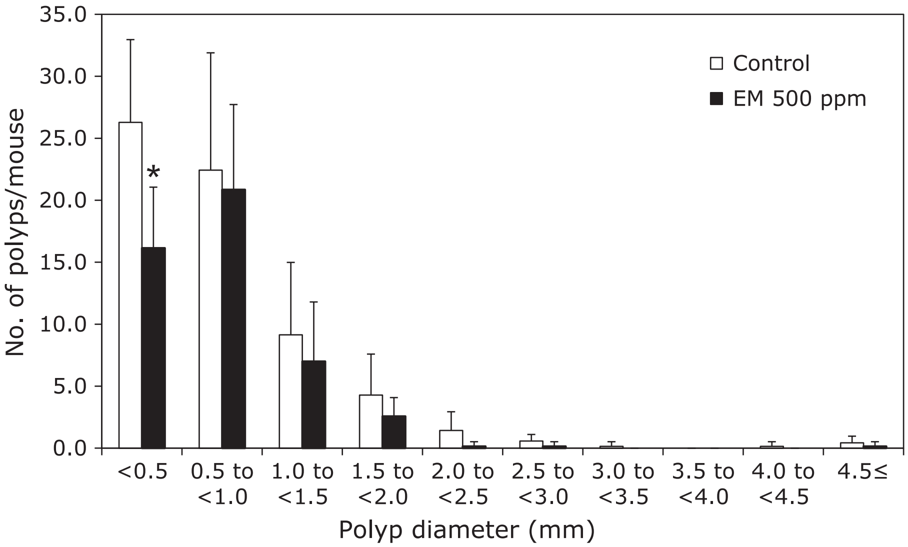

Supplemental Fig. 2. Min mice were fed a basal diet (open box) or a diet containing 500 ppm (black-filled box) erythromycin for 8 weeks. The number of polyps per mouse in each size class is given as the mean ± SD. *$p<0.05$ *vs* control 0 ppm.
