# Supplementary material for: Chemopreventive effects of a low-side-effect antibiotic drug, erythromycin, on mouse intestinal tumors
Source: J Clin Biochem Nutr. 2017 Apr 14;60(3):199–207. doi: 10.3164/jcbn.16-107 (PMC5453017; doi:10.3164/jcbn.16-107)
Supplement: Supplemental Fig. 3 [file jcbn16-107sf03.pdf]

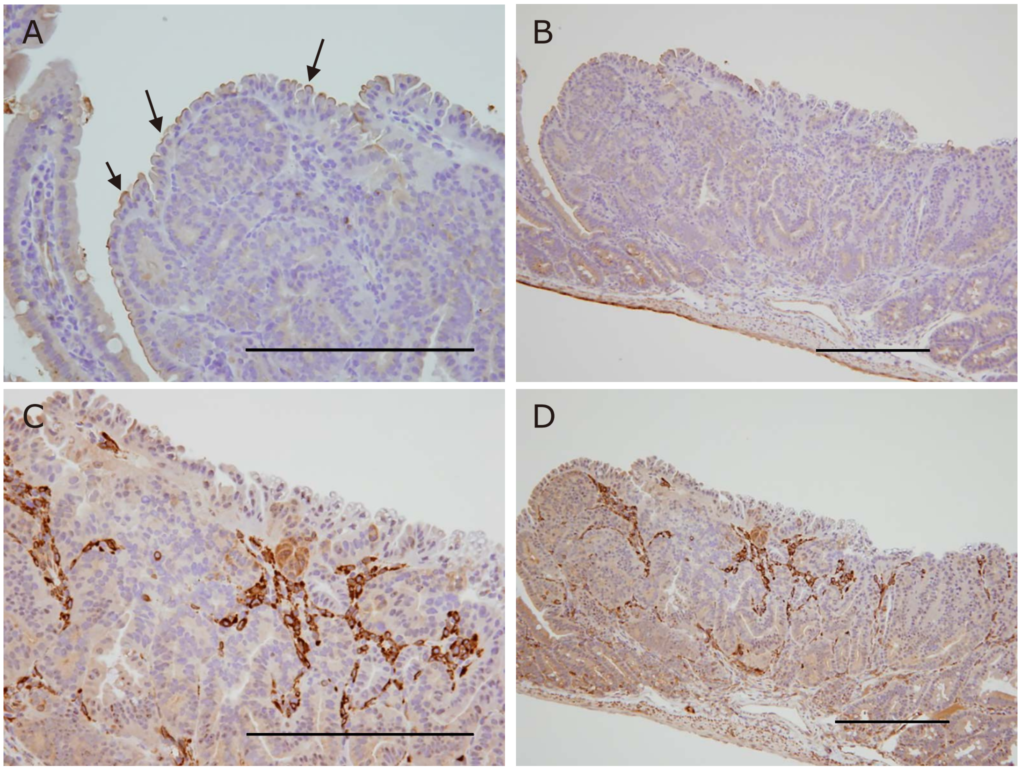

Supplemental Fig. 3. Immunohistochemical staining of IL-6 (A, B) and COX-2 (C, D) were localized and expressed in small intestinal polyps of Min mice, respectively. Scale bars = 100 μm. Arrow represents representative IL-6 positive cells.
